# Creating an Age-Friendly Public Health System

**DOI:** 10.1093/geroni/igz044

**Published:** 2020-01-01

**Authors:** Anne De Biasi, Megan Wolfe, Jane Carmody, Terry Fulmer, John Auerbach

**Affiliations:** 1 Trust for America’s Health, Washington, District of Columbia; 2 The John A. Hartford Foundation, New York

**Keywords:** Evidence-based practice, Longevity, Medicaid/Medicare, Organizational/institutional issues, Public health system, Social movement, Successful aging

## Abstract

**Background and Objectives:**

The public health system in America—at all levels—has relatively few specialized initiatives that prioritize the health and well-being of older adults. And when public health does address the needs of older adults, it is often as an afterthought. In consultation with leaders in public health, health care, and aging, an innovative *Framework for an Age-Friendly Public Health System* (Framework) was developed outlining roles that public health could fulfill, in collaboration with aging services, to address the challenges and opportunities of an aging society.

**Research Design and Methods:**

With leadership from Trust for America’s Health and The John A. Hartford Foundation, the Florida Departments of Health and Elder Affairs are piloting the implementation of this *Framework* within Florida’s county health departments and at the state level. The county health departments are expanding data collection efforts to identify older adult needs, creating new alliances with aging sector partners, coordinating with other agencies and community organizations to implement evidence-based programs and policies that address priority needs, and aligning efforts with the age-friendly communities and age-friendly health systems movements.

**Results, and Discussion and Implications:**

The county health departments in Florida participating in the pilot are leveraging the *Framework* to expand public health practice, programs, and policies that address health services and health behaviors, social, and economic factors and environmental conditions that allow older adults to age in place and live healthier and more productive lives. The model being piloted in Florida can be tailored to meet the unique needs of each community and their older adult population.

**Translational Significance:**

An age-friendly public health system is one that recognizes aging as a core public health issue and leverages its skills and capacities to improve the health and well-being of older adults. The impacts of the Florida pilot project to be described in the proposed article will be scalable in local and state health departments across the country. The value of this project is its flexibility in design and application, with multilevel policy and programmatic impacts through multisector collaboration.

Americans are living longer and more productive lives than ever before. The number of adults aged 65 and older increased by 33% over the past 10 years and is projected to nearly double by 2060 to 98 million, or 24% of the U.S. population (Administration on Aging & Administration for Community Living, 2018). The population of those ages 85 and older is also projected to grow from 6 million to 20 million by 2060. This is good news, as these older adults are also staying in the workforce, contributing their knowledge and skills, as well as their wisdom (Figure 1).

**Figure 1.**
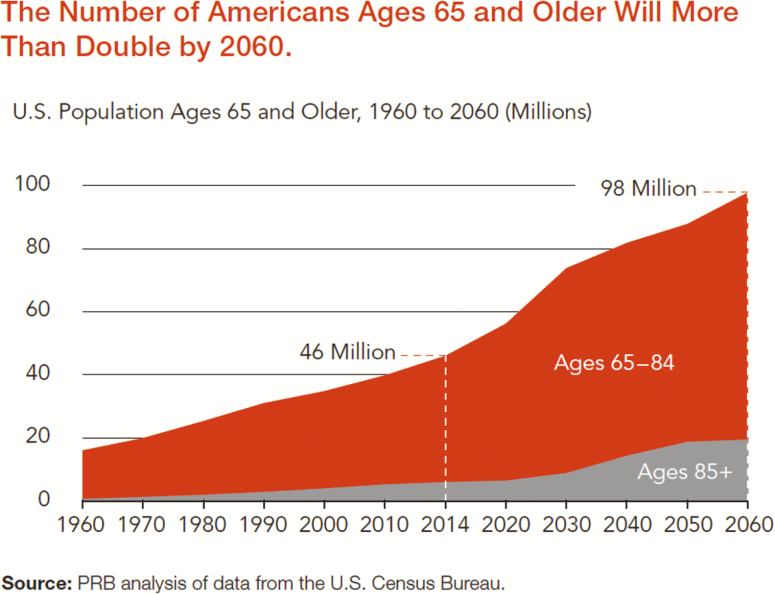
The number of Americans aged 65 and older will be more than double by 2060. *Source*: PRB analysis of data from the U.S. Census Bureau. (https://www.prb.org/wp-content/uploads/2016/01/aging-us-population-bulletin-1.pdf).

This rise in the number and proportion of older adults only presents part of the picture—the older adult population is becoming more racially and ethnically diverse. In 2014, 78% of older adults were non-Hispanic White, 9% were African American, 8% were Hispanic of any race, and 4% were Asian. By 2060, the percentage of non-Hispanic Whites is expected to drop to 55%, while the proportion of other racial groups will increase, with 22% of the population Hispanic, 12% African American, and 9% Asian (Federal Interagency Forum on Aging-Related Statistics, 2016). These changes are important because of racial and ethnic inequities in health and access to resources, as well as cultural differences in expectations of informal and formal care.

There are also substantial variations in social and economic well-being among the older adult population. Older adults are less likely to live below the poverty line than other age groups, with 10% of those ages 65 and over in poverty in 2014, but this may not be an accurate indicator of economic vulnerability in later life (Federal Interagency Forum on Aging-Related Statistics, 2016). The federal poverty level fails to consider all older adults’ basic living costs, including those for health care and transportation, and thus it underestimates the extent of financial need. Also, since poverty increases with age, the growth of the oldest-old may lead to an increase in the number of older adults living in poverty. Research using a more age-specific measure of financial resources found that in California more than half of older adults living alone and more than one-quarter of older couples lack adequate income to cover basic expenses (Wallace, Padilla-Frausto, & Smith, 2010).

Studies show that individuals subject to risk factors like racial, ethnic and socioeconomic disparities that accumulate over the life course are at greater risk for poor health and disease as older adults (Halfon & Hochstein, 2002). Given this and the increasing diversity along health and sociodemographic dimensions means that policies and programs designed to meet the needs of older adults must consider the needs and preferences of different subpopulations (Lehning & De Biasi, 2018). Health Affairs held a recent conference which highlighted that housing and health care costs for older adults in the “middle market” of income will soon be out of reach (Pearson et al., 2019).

Millions of older adults have chronic conditions, such as diabetes, hearing loss, arthritis, and heart disease (National Council on Aging, 2018). Eighty percent of Medicare beneficiaries (the insurance program for those ages 65 and older) have one chronic condition and nearly 70% have two or more (Centers for Disease Control and Prevention [CDC], 2011a). Chronic diseases can limit one’s ability to perform daily activities and result in a loss of independence, sometimes resulting in a need for institutional care, in-home caregivers, or other long-term services and supports (CDC, 2013). And chronic diseases are costly, accounting for two thirds of all health care costs and 93% of Medicare spending (Centers for Medicare and Medicaid Services, 2012; Gerteis et al., 2014). Yet, evidence shows that prevention is effective in reducing chronic diseases such as diabetes and heart disease and in preventing injuries (CDC, 2009). Programs such as the Medicare Diabetes Prevention Program have been found to reduce and prevent diabetes in Medicare beneficiaries with an indication of prediabetes (Centers for Medicare and Medicaid Services, 2017). And programs that promote physical activity in older adults can increase mobility and stability to help combat Alzheimer’s disease and avoid frailty and falls (Evidence Based Leadership Council, 2019). Despite the fact that health problems can be prevented, managed, or delayed with a stronger focus on prevention, in 2017, public health represented just 2.5% of all health spending in the country—or $274 per person (McKillip & Ilakkuvan, 2019).

Evidence also points to increased isolation and loneliness, with more older adults living away from their families, facing financial problems, having limited transportation and access to healthy food, and challenging housing options. Social isolation can negatively affect quality of life and contribute to an increased risk of morbidity and mortality (Bassuk, Glass, & Berkman, 1999). Research indicates loneliness can be more detrimental to health than smoking or obesity, increasing the risk for early death by 45% and the chance of developing dementia by 64% (Perissinotto, Cenzer & Covinsky, 2012; Worland, 2015). Social isolation has been estimated to account for $6.7 billion in additional Medicaid spending annually (AARP, 2018; Flowers et al., 2017).

According to a recent report by the U.S. Congressional Budget Office (CBO) (2019), federal spending on Medicare has been increasing since 2005. And overall, U.S. spending for people ages 65 and older has grown significantly; as a share of gross domestic product, mandatory spending for people in this age group grew from 5.8% in 2005 to 7.5% in 2018 ($1.3 trillion). CBO projects that share would grow to 9.8% ($2.7 trillion) by 2029 and that in 10 years, the federal government will spend half of its budget on mandatory programs for this population—Social Security and Medicare (CBO, 2019).

Despite these upward cost trends, growth of medical spending for older adults is actually slowing down, particularly for Medicare patients with chronic conditions (Cutler et al., 2019). Reduced spending on cardiovascular disease and risk factors (such as hypertension and diabetes) have been the biggest contributors to the spending slowdown (Cutler et al., 2019). This is due in part to the advances in science and resultant treatment that addresses heart disease and also to prevention programs that reduce risk factors related to heart disease (Stewart, Manmathan, & Wilkinson, 2017). Medically focused prevention, including preventive cardiovascular disease medications, also contributes to this spending slowdown (Figure 2).

**Figure 2.**
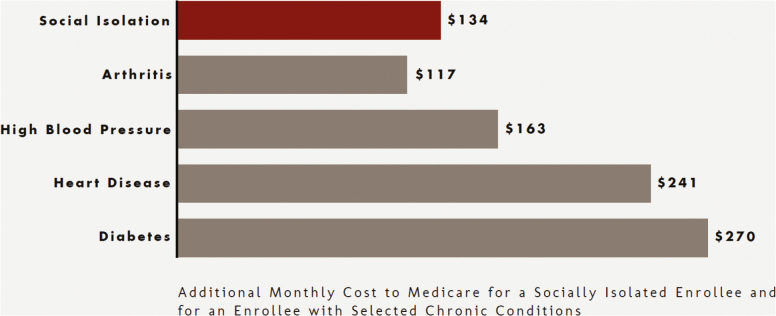
AARP: the costs of social isolation. (https://www.aarp.org/content/dam/aarp/ppi/2018/social-isolation-infographic.pdf).

Yet there is plenty of room for more prevention-related interventions that will lead to better outcomes for Medicare beneficiaries and even lower spending for the nation. Public health prevention programs that work to prevent disease and complications of disease, improve quality of life, and reduce health care costs include physical activity and nutrition programs, arthritis prevention and control, diabetes prevention and control, early detection of cancer, heart disease, and stroke prevention and chronic disease self-management programs (CDC, 2011b; Hoffman & Mertzlufft, 2018).

## Lack of a System Approach to Address Older Adult Health

Many national efforts have prioritized older adult health and well-being, including the Older Americans Act, the recent recognition of the importance of addressing social determinants in Medicare, national convenings such as the White House Conference on Aging and the U.S. Department of Health and Human Services (HHS) Healthy Aging Summit, and reports such as *Healthy Aging in Action* (National Prevention Council, 2016). Despite this national attention to the importance of health in our later years, programs that address older adult health continue to be siloed and under-resourced, and the United States is not making progress toward a system approach to improve the health and well-being of our older adults. The CDC has no staff dedicated solely to healthy aging programs. Other federal services are scattered across agencies and their leaders are not collaborating. Programs such as Age-Friendly Communities, CDC’s Healthy Brain Initiative, Dementia-Friendly Communities, Age-Friendly Health Systems, and many other efforts are in nascent stages, operating independently. There is a growing recognition that social and environmental factors affect health (Castrucci & Auerbach, 2019), and one of the most effective strategies to influence those factors is through the collective action approach (Tufts Health Plan Foundation, n.d.). However, national, state, and local stakeholders have not broadly adopted an aligned and coordinated older adult health and well-being approach.

## Lack of Public Health Engagement in Older Adult Health

Although public health efforts are largely responsible for the dramatic increases in longevity over the 20th century, there have been limited collaborations between the public health and aging sectors (Anderson, Goodman, Holtzman, Posner, & Northridge, 2012; Cutler & Miller, 2005). Older adults were not central to the public health agenda when public health emerged in cities in the 19th century (Kane, 1997). Similarly, in the mid-20th century, many policies designed to support older adult health and independence, including Medicare, Medicaid, and the Older Americans Act, did not explicitly include a role for public health. Over the past 50 years, some steps have been taken toward a more collaborative approach, such as the formation of the Aging and Public Health section of the American Public Health Association in 1978, or the mandated role for CDC in providing disease prevention and health promotion services offered through the Older Americans Act in 1987 (Anderson et al., 2012). However, public health agencies rarely have dedicated funding or initiatives targeting adults ages 65 and older.

In recent decades, the aging network, comprising 56 State Units on Aging, 655 Area Agencies on Aging (AAAs), 243 Indian Tribal and Native Hawaiian Organizations, and thousands of service providers and volunteers, has increasingly focused on prevention and wellness. The national association for AAAs, the National Association of Area Agencies on Aging (N4A), is currently working to build the capacity of AAAs to partner with health care providers and payers, like current public health partnership efforts. The 2010 passage of the Affordable Care Act (ACA) is shifting the health care system to one with a broadened focus on prevention, wellness, and health, rather than only disease. As mandated by the ACA, in 2011, the National Prevention Council released the National Prevention Strategy with an overarching goal of increasing the number of Americans who are healthy at every stage of life. In 2016, the Council produced *Healthy Aging in Action,* highlighting programs that are advancing the National Prevention Strategy specifically for older adults. Central to this report is the need for multisector collaborations to achieve a goal of healthy aging (National Prevention Council, 2016).

CDC has historically had a limited role in promoting the health and well-being of older adults. In 2001, CDC established the Prevention Research Centers Healthy Aging Research Network (PRC-HAN) to understand determinants of healthy aging and develop evidence-based community programs (Sleet, Moffett, & Stevens, 2008). The network consists of seven major universities and their affiliated communities, which collectively conduct research, develop and evaluate initiatives promoting healthy aging, and translate and disseminate research findings into evidence-based public health programs (Hunter et al., 2013). CDC has also supported effective falls prevention research and programs (Sleet et al., 2008). In 2005, CDC established the Healthy Brain Initiative to address Alzheimer’s disease and dementia, and in 2007, CDC published *The Healthy Brain Initiative: A National Public Health Road Map to Maintaining Cognitive Health (2007)*. This Road Map led to a better understanding of public’s perception of cognitive health as well as a revision of CDC’s Behavioral Risk Factor Surveillance System (BRFSS) to include questions about confusion, memory loss, and caregivers. The next iteration, *The Public Health Road Map for State and National Partnerships: 2013–2018*, emphasized the needs of caregivers and the importance of partnerships between public health and aging services professionals (Anderson & Egge, 2014).

## Public Health’s Potential Roles in Healthy Aging

Public health needs to be a critical partner in all efforts to support and promote programs that improve the health and well-being of older adults. Evidence shows that disease prevention and health promotion programs are effective, and they are the domain of public health (Keck School of Medicine, n.d.). Throughout the 20th century, public health played a crucial role in adding years to life. In the 21st century, public health can play a crucial role in adding life to years. Recognizing the significant role the U.S. public health system can play in strengthening older adult health, Trust for America’s Health (TFAH) led a convening in 2017, funded by The John A. Hartford Foundation, to explore potential roles for public health in healthy aging. National, state, and local public health officials, aging experts, advocates, and service providers, and health care officials who participated in the convening strongly endorsed a greater role for public health in aging. Through an examination of case studies of older adults, participants identified gaps in services, supports, and policies needed to improve older adult health and well-being and considered the potential roles public health could play in filling these identified gaps. The resulting *Framework for an Age-Friendly Public Health System* outlines the functions that public health could fulfill, in collaboration with aging services and the health care sector to address the challenges and opportunities of an aging society. The main takeaway from the convening was the need for an Age-Friendly Public Health (AFPH) system that recognizes aging as a core public health issue.

Healthy aging is defined in the *Framework* as (i) promoting health, preventing disease, injury, and frailty, and managing chronic conditions; (ii) optimizing physical, cognitive, and mental health; and (iii) facilitating social and civic engagement. This definition intentionally does not equate healthy aging with the absence of disease and disability. Instead, it portrays healthy aging as both an adaptive process in response to the challenges that can occur as we age, and a proactive process to reduce the likelihood, intensity, or impact of future challenges. Healthy aging involves maximizing physical, mental, emotional, and social well-being, while recognizing that aging is often accompanied by chronic illnesses and functional limitations. It also emphasizes the importance of meaningful involvement of older adults with others, such as friends, family members, neighbors, organizations, and the wider community. Although the public health sector has experience and skill in addressing these components of health for some populations, it has not traditionally focused attention on older adults.

The *Framework* is not a prescriptive guide to action or a declaration of the public health sector’s oversight of certain activities. Not every community will need public health to assume each of these roles. Agencies and organizations in other sectors are already actively engaged in healthy aging but are not leveraging the expertise of public health professionals. Public health should work in partnership with these organizations to promote healthy aging. Furthermore, public health organizations lack the resources to focus on healthy aging and will thus need to carefully and thoughtfully prioritize their roles. The *Framework* offers a useful articulation of the potential contributions that public health should consider as it embraces a larger role in optimizing the health of older adults.

**Figure 3.**
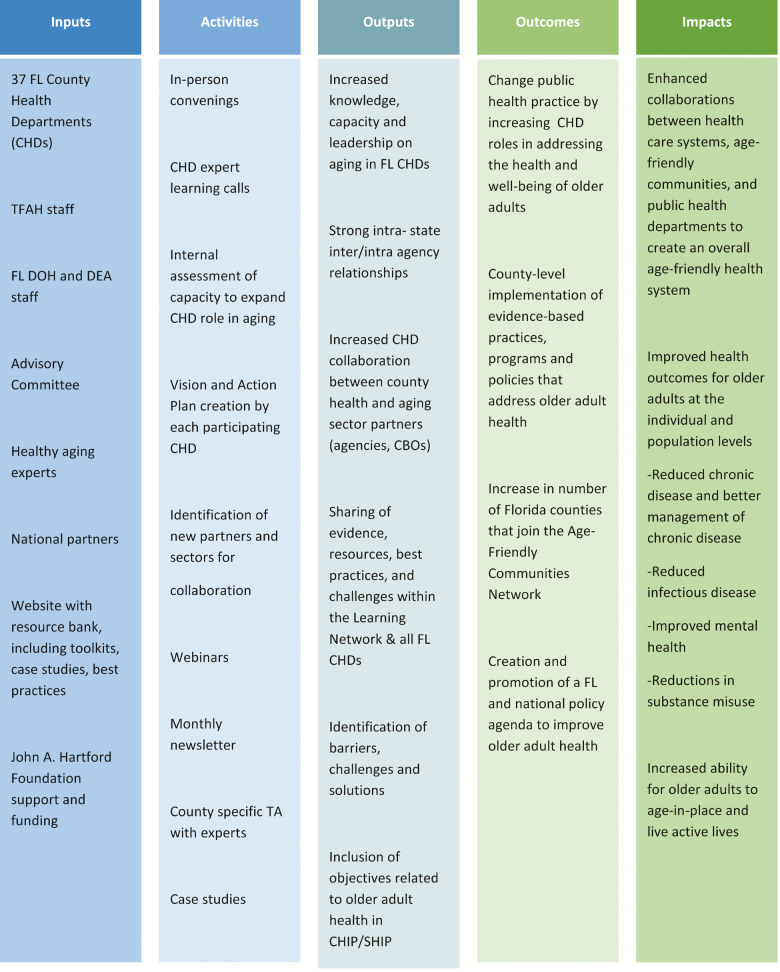
Trust for America’s health: age-friendly public health logic model.

To explore these functions more fully through actual public health experience, TFAH initiated the Florida-based Age-Friendly Public Health Learning and Action Network (AFPH Network), with funding from The John A. Hartford Foundation. TFAH created an application process to select county health departments (CHDs) to participate in the AFPH Network and conducted interviews with all prospective county teams. With support from the Florida Departments of Health and Elder Affairs, all applicants were invited to participate. The AFPH Network includes teams from 37 of Florida’s 67 CHDs, representing 65% of Florida’s overall population and 65% of the older adult population (Figure 3).

## Age-Friendly Health Systems’ Alignment With Age-Friendly Public Health

The Age-Friendly Health Systems movement, initiated in 2017, recognizes that an all-in, national response is needed to embrace the health and well-being of the growing older adult population. Like public health, health systems, including payers, hospitals, clinics, community-based organizations, nursing homes, and home health care, need to adopt a new way of thinking that replaces unwanted care and services with aligned interventions that respect older adults’ goals and preferences. Becoming an Age-Friendly Health System entails reliably acting on a set of four evidence-based elements of high-quality care and services, known as the “4Ms,” for all older adults. When implemented together, the 4Ms represent a broad shift to focus on the needs of older adults:

(1) What Matters: Know and align care with each older adult’s specific health outcome goals and care preferences including, but not limited to, end-of-life care and across settings of care;
(2) Medication: If medication is necessary, use Age-Friendly medication that does not interfere with What Matters to the older adult, Mobility, or Mentation across settings of care;
(3) Mentation: Prevent, identify, treat, and manage dementia, depression, and delirium across settings of care; and
(4) Mobility: Ensure that older adults move safely every day to maintain function and do What Matters (Mate, Berman, Laderman, Kabcenell, & Fulmer, 2018).

The initiative to advance AFPH systems strives to create community-wide conditions to improve the health and well-being of older adults that should be seamless across the continuum from clinical care to community. Health care systems are managing their identified populations and expanding their focus beyond their walls and into their communities by addressing the 4Ms with individual patients. The public health system works to create the social and environmental conditions that address the needs of the whole population of older adults associated with housing, food availability, social engagement, transportation, and safety, as well as by providing educational information and promoting healthier behaviors, such as better nutrition and more physical activity. Public health also implements evidence-based programs and policies to prevent disease, frailty, and cognitive decline. Public health can assist in community-based interventions to improve the social determinants of health for older adults and provide services unique to older populations (Ogden, Richards, & Shenson, 2012). Public health already serves in this role for some populations, but typically not for older adults (Figure 5).

## Age-Friendly Public Health Alignment with Age-Friendly Communities

**Figure 4.**
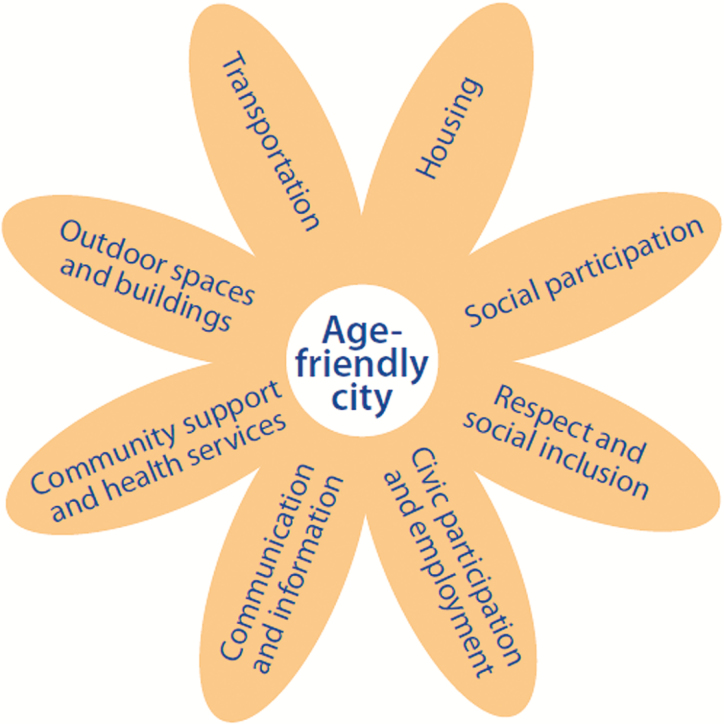
AARP: the domains of age-friendly communities.

In 2006, the World Health Organization (WHO) initiated a movement to create “Age-Friendly Communities,” those that encourage “active aging by optimizing opportunities for health, participation, and security in order to enhance quality of life as people age” (World Health Organization, 2007). Age-Friendly Communities (AFCs) typically focus on eight core community features that address the physical and social infrastructure to support the health across the life span: housing, transportation, social participation, respect and social inclusion, civic participation and employment, communication and information, community support and health services, and outdoor spaces and buildings (Alley, Liebig, Pynoos, Banerjee, & Choi, 2007) The AARP is the U.S. agent for the WHO AFC, and as of this writing, there are four states (Colorado, Massachusetts, Florida, and New York) and 368 U.S. cities and communities that have joined the AARP Network of Age-Friendly States and Communities (Figure 4).

**Figure 5.**
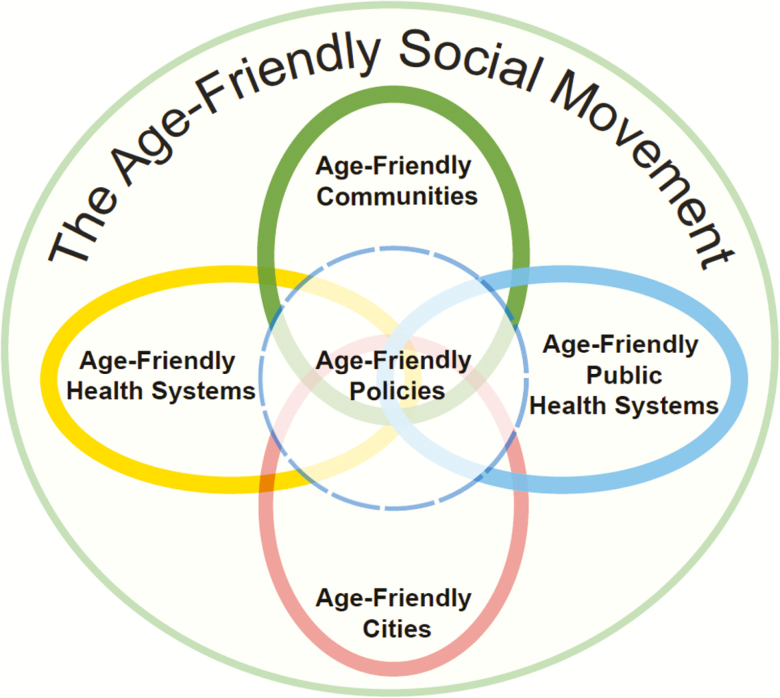
Used with permission from The John A. Hartford Foundation.

There is considerable alignment between the AFC domains and the social determinants of health, which are core to the work of public health, and public health can play an important role in helping to advance AFC initiatives and complement the associated policy and infrastructure changes that support community health (Figure 5).

## *Framework for an Age-Friendly Public Health System*: Five Roles for Public Health’s Engagement in Older Adult Health (Lehning & De Biasi, 2018)

Activities and opportunities that demonstrate the roles enumerated in the *Framework* are described in the following sections.

### Connecting and Convening Multiple Sectors and Professions That Provide the Supports, Services, and Infrastructure to Promote Healthy Aging

Addressing the full range of individual and community needs to support healthy aging requires the active contribution of a variety of stakeholders. As mentioned earlier, many different organizations and professionals are already working to improve older adult health and well-being, yet they operate in silos with limited opportunities to communicate and collaborate. The first role of public health is to connect and convene the multiple sectors and professions that provide the supports, services, and infrastructure to promote healthy aging.

One example that highlights this role is in promoting and supporting mobility and physical activity. Regular physical activity reduces the risk of chronic conditions, such as diabetes and cardiovascular disease, prevents cognitive and functional decline, and decreases the likelihood of falls and subsequent injury (Nelson et al., 2007); however, only a minority of older adults meet the recommendations outlined in the *2018 Physical Activity Guidelines for Americans* (U.S. Department of Health and Human Services, 2018). There are numerous barriers to regular physical activity in later life, including restricted access to indoor and outdoor recreational facilities, concerns about neighborhood safety, limited individual knowledge about the benefits of exercise (Schutzer & Graves, 2004), and the absence of walkable neighborhood features (e.g., well-maintained sidewalks, raised crosswalks, speed bumps, and a variety of food and shopping destinations; Clark, 1999). Public health could bring together the multiple actors needed to collaboratively address these barriers, including law enforcement, public works, parks and recreation, city planning, local businesses, health care systems, senior centers, and other community groups. Public health can promote communication across sectors, facilitate the sharing of knowledge and resources, including identifying relevant scientific evidence and effective evidence-based programs, and, in some cases, advance shared goal setting and action plans.

A second example is the need to address social isolation in later life. Social isolation can involve an objective separation from a social network, such as living alone, or more subjective feelings of loneliness (Golden et al., 2009). Approximately 12 million adults over the ages of 65 live alone (West, Cole, Goodkind, & He, 2014), and studies report that 15%–45% of older adults experience loneliness (Golden et al., 2009; Lauder, Sharkey, Mummery, 2004; Pinquart & Sorensen, 2001). Public health can work with community-based organizations to address loneliness and social isolation by providing opportunities for social interaction and the development of new friendships. For example, through the development of volunteer programs such as AARP’s Experience Corps, which engages older adults as volunteer tutors for young people in communities and schools (Martinez et al., 2006). Public health professionals can also partner with “Villages,” grassroots consumer-driven, community-based organizations that aim to promote aging-in-place by combining services, participant engagement, and peer support. First emerging in the early 2000s, currently, there are more than 200 Villages in the United States in operation or development (Village to Village Network, n.d.). Studies suggest that Villages are a promising approach to increasing members’ social engagement and connecting with a variety of formal and informal community supports (including those offered by public health departments) plays a crucial role in their ability to do so (Graham, Scharlach, & Price Wolf, 2014).

When convening sectors, professions, and organizations, public health typically leverages its seat at the table to ensure a focus on prevention, including policy, systems, and environmental change, to support population-level health improvement. A greater focus on prevention can help forestall declines in health and well-being, such as falls prevention and initiatives to promote physical activity or brain health. A focus on policy, systems, and environmental change complements the efforts to address the needs of individual older adults by focusing on improvements that affect entire populations or communities.

### Coordinating Existing Supports and Services to Avoid Duplication of Efforts, Identify Gaps, and Increase Access to Services and Supports

Navigating the wide variety of supports and services for older adults can be confusing and overwhelming. Supports and services are offered by a range of providers in different locations and settings, with different funding sources and variations in eligibility requirements. A second critical role for public health is, therefore, to coordinate existing supports and services to avoid duplication of efforts, identify gaps, and increase access. If resources are available, health departments can create an aging specialist role to facilitate this coordination and ensure that older adults are considered in any other public health programming or research.

Aging professionals and organizations, including AAAs, are already working to avoid duplication of efforts, reduce unmet need for supports, and maximize the efficient use of existing resources. Public health can be a particularly effective coordinator to address the barriers within its areas of expertise. For example, many older adults do not receive preventive health services, such as those recommended by the U.S. Preventive Services Task Force, including screenings, behavioral health monitoring and counseling, and immunizations (Office of Disease Prevention and Health Promotion, n.d.). With 90% of flu-related deaths occurring among those ages 65 and older, there is a critical need to improve access to vaccines (Halloran, 2013). Public health has been a key partner in the work of Vote & Vax, a national initiative that has received support from CDC and the Robert Wood Johnson Foundation to provide flu vaccines in polling places. Bringing together multiple sectors, including public health, pharmacy, and nursing, Vote & Vax has demonstrated success in improving vaccination rates among those with access barriers to the more traditional vaccination sites of physician offices or pharmacies (Shenson, Moore, Benson, & Anderson, 2015). This program thus fills a critical gap in service delivery and highlights a creative approach to improving population health, leveraging public health’s traditional role of working to increase access to clinical preventive services.

### Collecting Data to Assess Community Health Status (Including Inequities) and Aging Population Needs to Inform the Development of Interventions

All sectors are becoming increasingly data driven to ensure they have all the information they need to address their target populations and most pressing problems. A third role for public health is to call attention to the health status, needs and assets of a community’s aging population to inform their community health needs assessment (CHNA), a critical step to set goals and implement strategies for health improvement. Public health can document health status by collecting and analyzing data, including data from multiple sectors and sources.

In response to the needs of the Florida AFPH Network CHDs for data on the health of the older adults in their counties, the Florida Department of Health, in collaboration with the Florida Department of Elder Affairs, created new “Aging in Florida” health profiles. Each participating CHD is now analyzing their county data to assess the health status and needs of older adults, with a particular focus on improving health equity. Each AFPH Network team has analyzed the data, prioritized the needs and opportunities, and developed an action plan.

CDC’s BRFSS as noted above was augmented to include two voluntary modules for states to assess cognitive decline and caregiver health. These two modules are in use in 35 states (with 21 states using the caregiver module, 21 states measuring cognitive decline, and 7 states implementing both). Public health departments can advocate for wider implementation of these modules and can analyze and disseminate the data in states that have implemented one or both.

Public health can also provide important information about older adults using hotspot analysis, a technique to examine the geographic distribution of populations, features, or events. Geographic data can be essential in mapping neighborhoods where older adults are at a higher risk for falls or have less access to a grocery store. Hotspot analyses showing areas with high concentrations of older adults, particularly those living alone or with a health challenge, could also enhance emergency preparedness planning, which is critical because older adults often experience higher rates of injury and death and lower rates of economic recovery following major natural disasters (Bolin, & Klenow, 1983). The Department of Health and Human Services’ Office of the Assistant Secretary for Preparedness and Response developed the emPOWER Initiative through a partnership with the Centers for Medicare and Medicaid Services. This initiative provides federal data and mapping tools to public health departments to help identify vulnerable populations who rely on electricity-dependent medical and assistive devices or certain health care services such as dialysis machines, oxygen tanks, and home health services. The emPOWER Map is a public, interactive map that provides monthly de-identified Medicare data down to the ZIP code level, and near real-time hazard tracking services. Together, this information provides enhanced situational awareness and actionable information for assisting areas and at-risk populations that may be impacted by severe weather, wildfires, earthquakes, and other disasters. Public health and emergency management officials, AAAs, and community planners can use emPOWER to better understand the types of resources that may be needed in an emergency. For instance, these data can inform power restoration prioritization efforts, identify optimal locations for shelters, determine transportation needs, and anticipate potential emergency medical assistance requests. The data are also used to conduct outreach to at-risk older adult populations prior to, during, or after an emergency.

Public health can also provide hospitals and health systems with information about their local older adult population in their surrounding communities as part of CHNAs, which are required every 3 years for all tax-exempt hospitals, to assess and prioritize the health needs of their geographic community and develop and implement action steps to address those needs (Office for State, Tribal, Local, and Territorial Support, Public Health Law Program, 2013). At least one local, state, or regional public health department must be involved in this process. Public health can thus call attention to the needs of older adults and ensure programs and resources are dedicated to this population.

### Conducting, Communicating, and Disseminating Research Findings and Best Practices to Support Healthy Aging

Public health researchers, policymakers, and practitioners can also play key roles in supporting healthy aging by conducting, communicating, and disseminating research findings and best practices to empower individuals to engage in healthy behaviors; support the provision of effective services; and contribute to the creation of safe and healthy community environments. There is a large body of research concerning healthy aging, yet limited clearinghouses for interested parties to find best practices or resources. Public health plays a key role in translating research findings into practice through education of providers and providing implementation support. Public health organizations could serve as central locations for best practices, toolkits, and research on healthy aging. The ready availability of this information would enhance the capacity of other sectors and professions to address the needs of older adults.

Public health is already serving this function in the area of cognitive health. Approximately 10%, or 3.6 million, of all Medicare beneficiaries over the age of 65 living in the community had some form of dementia in 2011 (Federal Interagency Forum on Aging-Related Statistics, 2016). CDC’s Healthy Brain Initiative promotes a role for public health in maintaining or improving cognitive functioning in later life. As part of this initiative, CDC and the Alzheimer’s Association developed a guide as noted above outlining strategies for public health to promote cognitive health, address cognitive impairment, and support dementia caregivers (Alzheimer’s Association & CDC, 2013). A key component of this initiative is supporting applied research and translating evidence into practice. Public health also assists with neurocognitive disorder public awareness campaigns around modifiable risk factors, signs of disease progression, strategies for addressing changes in behavior, and community supports.

### Complementing and Supplementing Existing Supports and Services, Particularly in Terms of Integrating Clinical and Population Health Approaches

The fifth role for public health is complementing and supplementing existing supports and services in terms of integrating clinical and population health approaches. Existing public health programs address a wide range of health issues, from infectious disease to chronic disease; from education campaigns that reach the general public, to targeted and focused home visits by educators; from the enforcement of environmental regulations addressing long-term health risks, such as lack of clean air and water, to the response to rare and catastrophic events. Furthermore, public health is focused on the entire life course, providing programs and policies, such as maternal and child health, workplace safety, and tobacco-free initiatives, that ultimately support healthy aging later in life. Each of these current activities could be assessed to determine whether it is adequately meeting the needs of older adults and, when necessary, modified to better do so. For example, aging services are beginning to recognize the value of community health workers (CHWs), a public health approach that has long been working with populations with limited access to formal health and social services. CHWs are trusted members of a community and conduct outreach, provide education, and serve as a liaison to formal systems of support. Preliminary research indicates the promise of CHWs for reducing health care costs, supporting transitions back home from the hospital, and connecting low-income senior housing residents to community services (Rush, 2019). This may be a particularly effective strategy to address health inequities and to address social isolation.

Public health can also complement existing programs for informal caregivers that provide assistance to older adults with disabilities. Community-based supports for caregivers are often fragmented from each other and disconnected from the health and long-term care systems (Riggs, 2003). The National Family Caregiver Support Program provides a range of services, including counseling, case management, respite care, and training, particularly in terms of adapting to the caregiver role and developing strategies for self-care, yet gaps remain. Public health can provide critical education and training on performing the tasks needed to support older care recipients, such as safely bathing or transferring from a bed to a chair, or addressing the behavioral changes associated with dementia.

### Conclusion

Public health’s mission is to improve the health and safety of our nation. Yet public health is not adequately engaged in efforts to improve the health and well-being of the older adult population, despite the overwhelming evidence of the effectiveness of prevention and health promotion activities that improve older adult health and quality of life. TFAH, with support from The John A. Hartford Foundation, developed a *Framework* outlining the key roles that public health can fulfill, in alignment with partners in aging and other sectors, to modernize the public health system by translating evidence on healthy aging into public health practice. Increasing the engagement of local, state, and federal public health offers promise to improve the health and well-being of this population—a precious resource for our nation—and bend the rising cost curve by investing more in prevention.
